# Anoikis-related gene signature is associated with immune infiltration and predicts the prognosis of non-small cell lung cancer

**DOI:** 10.18632/aging.205522

**Published:** 2024-02-07

**Authors:** Yixuan Wu, Zhou Zhou, Qianyi Qi, Shirong Xu, Lin Chen, Feng Wang

**Affiliations:** 1 Research Center of Clinical Medicine, Affiliated Hospital of Nantong University, Medical School of Nantong University, Nantong 226001, China; 2 Department of Laboratory Medicine, Affiliated Hospital of Nantong University, Medical School of Nantong University, Nantong 226001, China; 3 Department of Laboratory Medicine, Taizhou Second People’s Hospital, Taizhou 225511, China; 4 Nantong Institute of Liver Diseases, Nantong Third People’s Hospital Affiliated Nantong Hospital 3 of Nantong University, Nantong 226006, China

**Keywords:** NSCLC, anoikis, gene signature, HMGA1, immunotherapy

## Abstract

Non-small cell lung cancer (NSCLC) is the most common histological type of lung cancer. With the in-depth exploration of cell death manners, numerous studies found that anoikis is an important mechanism that associated with treatment. Therefore, we aimed to explore the prognostic value and treatment guidance of anoikis in NSCLC patients. In the current study, we first constructed a prognostic model based on the anoikis-related genes based on bulk RNA-sequencing and single-cell RNA-sequencing (scRNA-seq) dataset. Then, immuno-correlations of anoikis-related risk scores (ARGRS) were analyzed. In addition, HMGA1, a risky gene in ARGRS, was further explored to define its expression and immuno-correlation. Results showed that patients with higher ARGRS had worse clinical outcomes. Moreover, the five genes in the prognostic model were all highly expressed on tumor cells. Moreover, further analysis found that the ARGRS was negatively correlated with ImmuneScore, but positively with tumor purity. Besides, patients in the ARGRS-high group had lower levels of immunological characteristics, such as the immune-related signaling pathways and subpopulations. Additionally, in the immunotherapy cohorts, patients with the ARGRS-high phenotype were more resistant to immunotherapy and tended to not achieve remission after treatment. Last, HMGA1 was chosen as the representative biomarker, and analysis of the in-house cohort showed that HMGA1 was highly expressed in tumor tissues and correlated with decreased T cell infiltration. To sum up, ARGRS was correlated with a desert tumor microenvironment and identified immune-cold tumors, which can be a novel biomarker for the recognition of immunological characteristics and an immunotherapeutic response in NSCLC.

## INTRODUCTION

With over 1,600,000 newly diagnosed patients each year, lung cancer is an extraordinarily heterogeneous illness and the acknowledged leading cause of the majority of cancer-related mortalities globally [1]. Non-small cell lung cancer (NSCLC), containing lung adenocarcinoma (LUAD) and lung squamous cell carcinoma (LUSC) [2], is the most common histological type of lung cancer, accounting for around 85% of all occurrences [3]. Despite tremendous advances in clinical screening and therapeutic therapies for NSCLC, the limited therapeutic benefit of first-line treatment resulted in a low overall cure and survival rate for NSCLC, particularly in metastatic disease. Despite tremendous advances in clinical screening and therapeutic therapies for NSCLC, the limited therapeutic benefit of first-line treatment resulted in a low overall cure and survival rate for NSCLC, particularly in metastatic disease [4]. As a result, additional research is needed to uncover more relevant biomarkers in order to extend clinical advantages to a larger patient population and enhance NSCLC outcomes.

Immunotherapy, which strengthens the patient’s immune system to fight malignancies, has received massive attention in recent years in the context of cancer treatment. Immunosurveillance, or the ability of immune cells in the tumor microenvironment (TME) to recognize and destroy cancer cells under normal conditions, is now widely acknowledged [5, 6]. Nevertheless, further investigation showed that cancer cells can control the host immune system to avoid immune monitoring by enlisting immunosuppressive cell populations and reducing the immunogenicity of tumors [7, 8]. In addition, tumors with different phenotype have distinct therapeutic responses. To be specific, hot tumors, featured by T-cell inflammation, showed a favorite therapeutic response to immunotherapy, while cold tumors are resistant to many treatments [9–12]. Thus, it is crucial to investigate the alteration of the TME to guide the personalized immunotherapy.

In the recent years, with the intensive investigation of different manners of cell death, numerous researchers found that anoikis is an important mechanism that can be introduced to treatment. Anoikis is a type of programmed cell death that takes place when cells separate from the proper extracellular matrix. This mechanism is essential for maintaining plastic cell development and attachment [13]. Notably, cancer cells are resistant to anoikis because they do not depend on extracellular matrix adherence for survival and growth [14], indicating that malignancies are a better example of anoikis resistance. Therefore, understanding the NSCLC anoikis regulators helps researchers find new treatments, particularly for cancer metastasis [15, 16]. For instance, through altering the JAK2/STAT3 and SHP2/Grb2 signaling pathways, the TGF-1/SH2B3 axis can control lung cancer cells’ anoikis resistance and EMT [17]. In addition, the enhancement of anoikis sensitivity could enhance immune cell-mediated cytotoxicity [18]. However, the association between anoikis and TME features in NSCLC is still unclear.

At present, several studies explored the correlation between the anoikis feature and immunological characteristics. Here, in this study, we first recognized the up-regulated anoikis-related genes (ARG) in NSCLC tumor tissues, and then construct a prognostic model based on these genes. Subsequently, we confirmed that the ARGs in the prognostic model were expressed on tumor cells at the single-cell level. Finally, further analysis was performed to explore the correlation between ARGs and immunological characteristics and the predictive value of ARGs model in immunotherapeutic response. Taken together, our study provided a new perspective to understand the clinical and immunological-related functions of ARGs, which contributes to the advancement of more precise and precise treatment strategies.

## MATERIALS AND METHODS

### Data collection and processing

The gene expression matrices of NSCLC patients were downloaded from public online databases —the UCSC Xena website and the Gene Expression Omnibus (GEO) portal. The transcriptional omics and clinical annotations of tumors and paracancerous of NSCLC patients in the TCGA-LUAD and TCGA-LUSC cohorts were obtained from the UCSC Xena. In the GEO database, we identified three NSCLC cohorts (GSE30219 [19], GSE37745 [20], and GSE3141 [21]) with prognosis information. Besides, two clinical cohorts (GSE126044 [22] and GSE135222 [23]) of NSCLC patients received immunotherapy, were also obtained from the GEO database. Diagnostic patients with follow-up information, including survival outcomes or therapeutic responses were chosen for further analysis.

### Establishment of the anoikis-related gene model

To establish the anoikis-related gene (ARGs) model, we collected the ARGs from the the genecards website (https://www.genecards.org/, accessed on 12 October 2022) [24] and the Harmonizome portals (https://maayanlab.cloud/Harmonizome/, accessed on 12 October 2022) [25] firstly. Then, after performing differential expressions analysis by “limma” package, ARGs up-regulated in tumor samples (fold-change (FC) ≥ 1.5 and adjusted P-values < 0.05) were selected. The univariable COX regression analysis was used to identify genes that were significantly (P-value < 0.05) linked to OS. Next, the least absolute shrinkage and selection operator (LASSO) regression algorithm was performed on these OS-related genes to further screen prognostic parameters and construct the model. The risk score of the prognostic model based on NSCLC-related ARGs (ARGRS) of patients was assess according to the linear combination of the expression values of NSCLC-related ARGs multiplied by the relevant LASSO coefficients. To validate the risk score’s predictive power, the 50% ARGRS cutoff was used to divide the NSCLC patients into high- and ARGRS-low groups.

### Assessment of biological functions

The R package “clusterProfiler” [26] was used to assess the biological functions of gene signatures in terms of Gene Ontology (GO) [27] and Kyoto Encyclopedia of Genes and Genomes (KEGG) [28] pathways. The top ten enriched pathways with the most significantly P-values were displayed.

### Single-cell RNA sequencing datasets analysis

To further elaborate the tumor-specific of gene signatures in the prognostic model, single-cell RNA sequencing (scRNA-seq) datasets (GSE150660 [29] and GSE148071 [30]) were downloaded from the GEO protocol (All additional analyses were using the Seurat R toolkit [31].

For each sample, we summarized the expressed percentage of mitochondrial genes (percent.mt). Cells with percent.mt < 10% and 200 < nFeature_RNA < 5,000 were preserved. Then, for each dataset, the “RunHarmony” function [32] was applied to minimize the batch effects and integrate the transcriptional gene profiles from different patients. Principal component analysis (PCA) was performed based on the top 4,000 genes with highest variability. Then, the high dimensionality of data was reduced based on the top 30 PCs. The cells were unsupervised clustered via shared nearest neighbor (SNN) algorithm with one resolution. To annotate cell types, many well-known signatures were collected from previous studies [33, 34], such as EPCAM for tumor cells, CD3E for T cells, CD14 for macrophages, and CD1C for dendritic cells.

### Cell-cell communication analysis

“CellPhoneDB” software [35] was utilized to deconstruct the cell-cell communications among cell types at the single cell level. To define the ARGRS+ and ARGRS- tumor cells, “AddModuleScore” function was applied to estimate the enrichment scores of the ARGRS based on the transcriptional level of the five genes in the model. Then, tumor cells with ARGRS > 0 were defined as ARGRS+ tumor cells, and other were ARGRS- tumor cells. The ligand-receptor pairs with P-value < 0.05 were included in further analysis.

### Immune infiltration analysis

The gene signatures of immunomodulators, immune cell subpopulations and immunological signaling pathways were obtained from previous studies [36–38]. R package “estimate” was employed to examine the abundance of infiltrating immune cells (ImmuneScore) and tumor purity (TumorPurity). The R package “GSVA” (version 1.46.0) [39] was utilized to assess the enrichment scores of characteristics based on the gene signatures.

### Clinical samples, immunohistochemistry, and quantitative evaluation

The NSCLC tissue microarray (TMA) section (HLugC120PT01) was obtained from Outdo Biotech (Shanghai, China). Ethical approval (YB-M-05-02) for the study of the TMA was granted by the Clinical Research Ethics Committee, Outdo Biotech (Shanghai, China). The TMA was used for immunohistochemistry (IHC) assay to measure the expression of HMGA1 protein in tumor and non-tumor breast tissues. IHC assay was performed on these sections according to the established steps. These sections were given three 5-minute xylene washes and rehydrated using a series of washes in ethanol grades of 70%, 90%, and 100%. For twenty minutes, endogenous peroxidase activity was inhibited using hydrogen peroxidase. The antigen retrieval solution is EDTA. The primary antibody utilized in the study was anti-HMGA1 (1:200 dilution, Cat. sc-393213, Santa Cruz) and anti-CD8A (ready-to-use, Cat. PA577, Abcarta). Antibody staining was visualized with DAB and hematoxylin counterstain. Using the immunoreactivity score standard, two senior pathologists assessed stained TMA to determine HMGA1 expression [40]. Two senior pathologists used The Cancer Genome Atlas Network’s criteria to determine the CD8 score for tumor-infiltrating CD8^+^ T cell assessment [41]. For every sample, a CD8 score, which is the total of the density and distribution scores (0–6), was determined. Samples classified as immune-cold are those with a CD8 score ≤ 2 (0, 2) and immune-hot samples with a score ≥ 3 (3, 4, 5, 6).

### Statistical analysis

R-4.2.2 was used to perform all statistical analyses. The chi-square test was used to compare categorical variables, while the Wilcoxon rank-sum test was for continuous variables between the two groups. The log-rank test was used to assess the prognostic values. A two-paired p-value of less than 0.05 was considered statistically significant for all analyses, and the results were categorized as follows: *p-value < 0.05, **p-value ≤ 0.01, ***p-value ≤ 0.001, and ****p-value ≤ 0.0001.

### Availability of data and materials

The TCGA data are openly available at https://portal.gdc.cancer.gov/, and the GEO data are openly available at https://www.ncbi.nlm.nih.gov/gds.

### Consent for publication

All authors are consent for publication.

## RESULTS

### Identification of differentially expressed overlapping anoikis-related genes

**Figure 1:**
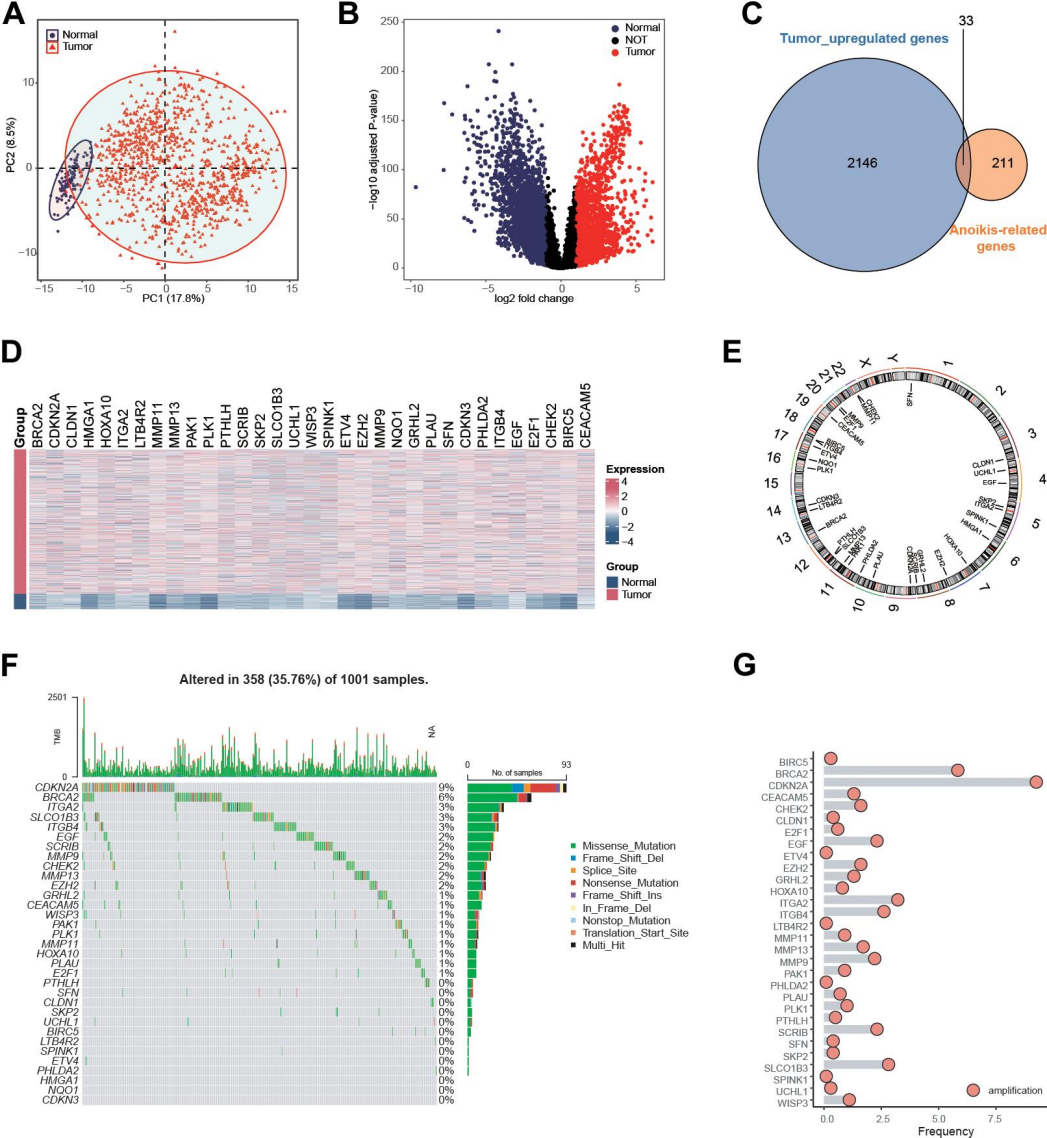
**Identification of anoikis-related genes in NSCLC patients in the TCGA NSCLC cohort.** (**A**) Principal component analysis of TCGA samples based on the expression matrix of anoikis-related genes obtained from the genecards website and the Harmonizome portals. (**B**) Volcano plot showing the differentially expressed genes (DEGs) for the NSCLC tissues and paracancerous in the TCGA-NSCLC cohort. (**C**) 33 anoikis-related genes were up-regulated in NSCLC patients in the TCGA cohort. (**D**) Heatmap showing the expression values of anoikis-related between the NSCLC tissues and paracancerous in the TCGA-NSCLC cohort. (**E**) The specific location of anoikis-related genes on the human chromosomes. (**F**) Mutation frequency of 33 overlapping signature genes in the TCGA NSCLC cohort. Each column represented a single patient. The upper bar plot showed TMB. The number on the right indicates the mutation frequency of each regulatory gene. The right bar plot showed the proportion of each variant type. The stacked bar plot below showed a fraction of conversions in each sample. (**G**) CNV frequency of 33 overlapping genes in the TCGA NSCLC cohort. The height of the column represented the alteration frequency. Blue dot: the deletion frequency; Red dot: the amplification frequency.

From the genecards websiteand the Harmonizome portals, a total of 244 anoikis-related genes (ARGs) were obtained. By performing PCA on the transcriptional matrix of anoikis-related genes, the normal samples and NSCLC patients clearly differed (Figure 1A). Therefore, a differential expression analysis was carried out to better identify NSCLC patients who had anoikis (Figure 1B). 33 anoikis-related genes were shown to be up-regulated in NSCLC patients, according to the results (Figure 1C–1E). Additionally, given the correlation between genomic variants and tumorigenesis, we summarized the mutation rate and copy number variation (CNV) of these genes. Results showed most of genes carried genomic mutations or amplifications in NSCLC patients (Figure 1F–1G), indicating the potential malignant functions of these in tumorigenesis.

### Establishment of a prognostic model of anoikis-related genes in NSCLC

**Figure 2:**
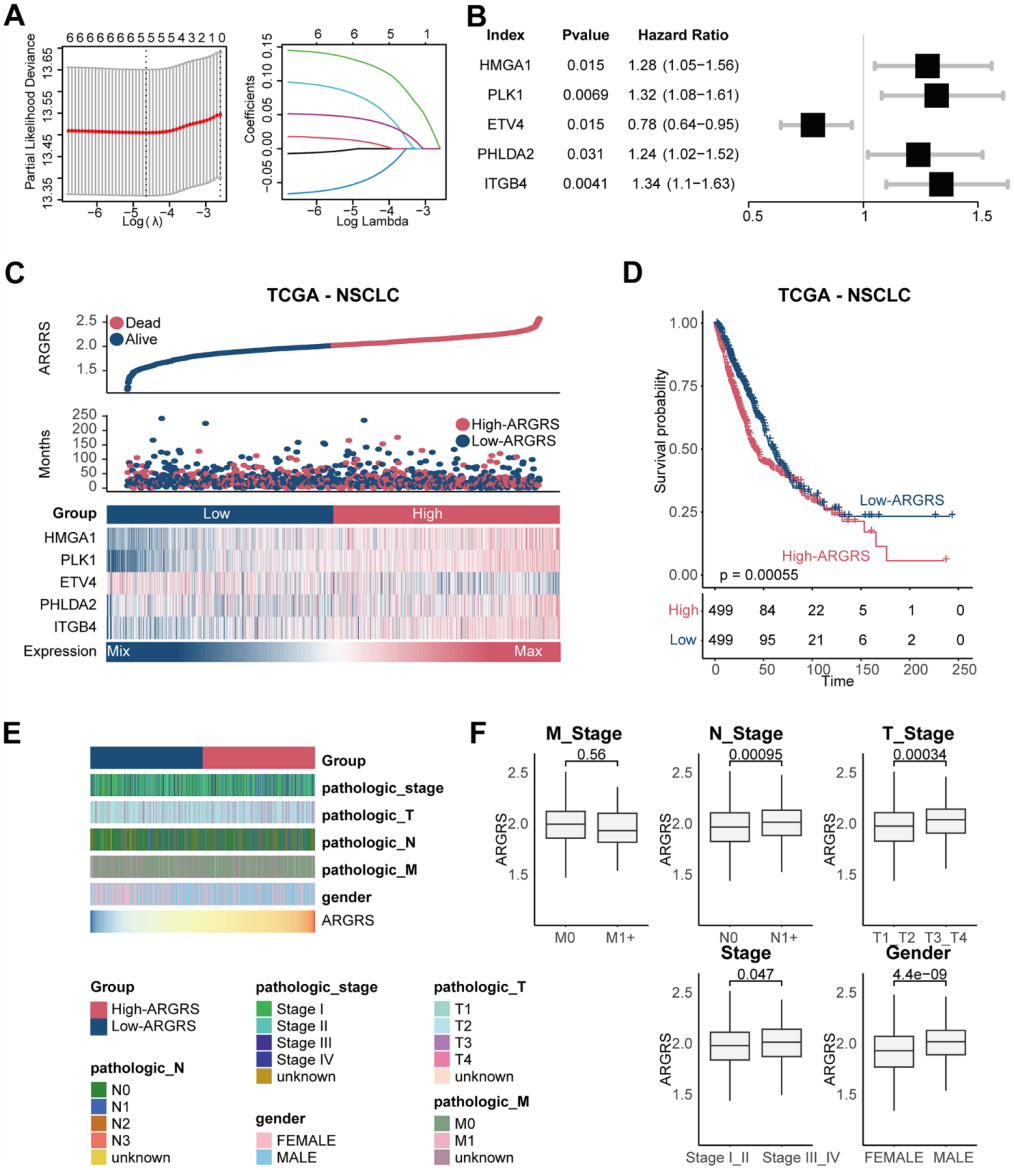
**Construction of prognostic model in the TCGA NSCLC cohort.** (**A**) The LASSO coefficient profiles were constructed using the 33 anoikis-related genes, and the tuning parameter (λ) was calculated based on the minimum criteria for OS with ten-fold cross validation. Five genes were selected according to the best fit profile. (**B**) Univariable analyses of the expression values of the five genes with overall survival in the TCGA NSCLC cohort. (**C**) Distributions of ARGRS, survival status of NSCLC patients, and expression profiles of the gene signatures. (**D**) Survival analysis showing the prognostic value of ARGRS in the TCGA NSCLC cohort. (**E**) Heatmap showing the distribution of clinicopathologic features between ARGRS-high and low groups. (**F**) Comparison of ARGRS among different clinicopathologic features.

By performing univariable COX regression analysis and LASSO analysis, five independent prognostic genes (HMGA1, PLK1, ETV4, PHLDA2, and ITGB4) were screened for establishing the prognostic model (Figure 2A–2B and Supplementary Figure 1). The risk scores based on the five anoikis-related genes (ARGRS) were calculated according to the combination of the expression levels of these genes multiplied by the corresponding coefficients. As shown in Figure 2C, the mortalities were centralized in the ARGRS-high group, while the living patients were enriched in the ARGRS-low group. Besides, in patients with higher ARGRS, signatures (HMGA1, PLK1, PHLDA2, and ITGB4) associated with poor OS were significantly highly expressed, while ETV4, correlated with favorable clinical outcomes showed the opposite (Figure 2C). Consistently, compared with the ARGRS-low group, patients with high ARGRS showed a worse prognosis (Figure 2D). In addition, further analysis showed that patients with higher pathological stages or grades had higher levels of signatures in the prognostic model and ARGRS (Figure 2E, 2F and Supplementary Figure 2), which further supported the finding that ARGRS was associated with worse clinical outcomes in NSCLC.

In addition, due to the consequence of genomic alterations in diagnosis and therapeutic guidelines, we also compared the mutant frequencies of genes between the high and low ARGRS groups. Compared with the ARGRS-low group, the ARGRS-high group had more complex genomic mutations (Supplementary Figure 3). Some well-known mutations associated with tumorigenesis and progression, such as TP53 [42, 43] and PTEN [44], were enriched in the ARGRS-high group, further supporting the association between ARGRS and worse clinical outcomes in NSCLC.

### Validation of the prognostic model in NSCLC

**Figure 3:**
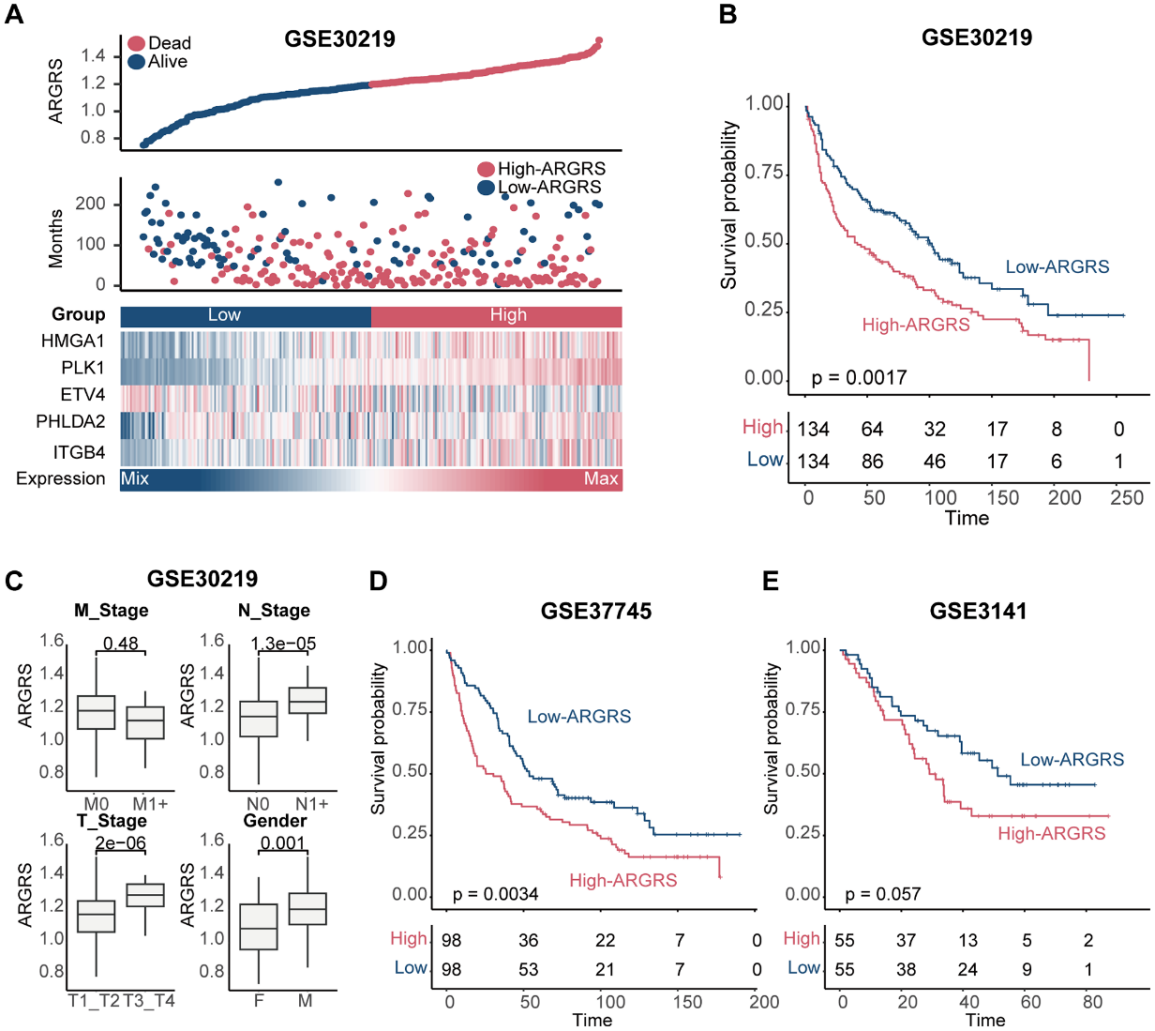
**Validation of prognostic model in other independent cohorts.** (**A**) Distributions of ARGRS, survival status of NSCLC patients, and expression profiles of the gene signatures in the GSE30219 cohort. (**B**) Survival analysis showing the prognostic value of ARGRS in the GSE30219 cohort. (**C**) Comparison of ARGRS among different clinicopathologic features. (**D**, **E**) Survival analysis showing the prognostic value of ARGRS in the (**D**) GSE37745 and (**E**) GSE3141 cohort.

We further confirmed this finding in additional independent cohorts after discovering the prognostic predictive utility of ARGRS in the TCGA cohort. Patients in the GSE30219 cohort who had higher ARGRS had worse OS than those with lower ARGRS, which is consistent with the findings from the TCGA cohort (Figure 3A, 3B). ARGRS levels were also higher in patients with higher clinical stages and grades (Figure 3C), indicating its role in the malignancy of NSCLC. Patients in the ARGRS-high group similarly exhibited inferior clinical outcomes in the GSE37745 and the GSE3141 cohorts (Figure 3D, 3E). Based on the findings from the TCGA and additional independent cohorts, the ARGRS was linked to a worse OS for NSCLC patients and may have contributed to tumor progression.

### The five anoikis-related genes in the model expressed specifically in tumor cells at the single-cell level

**Figure 4:**
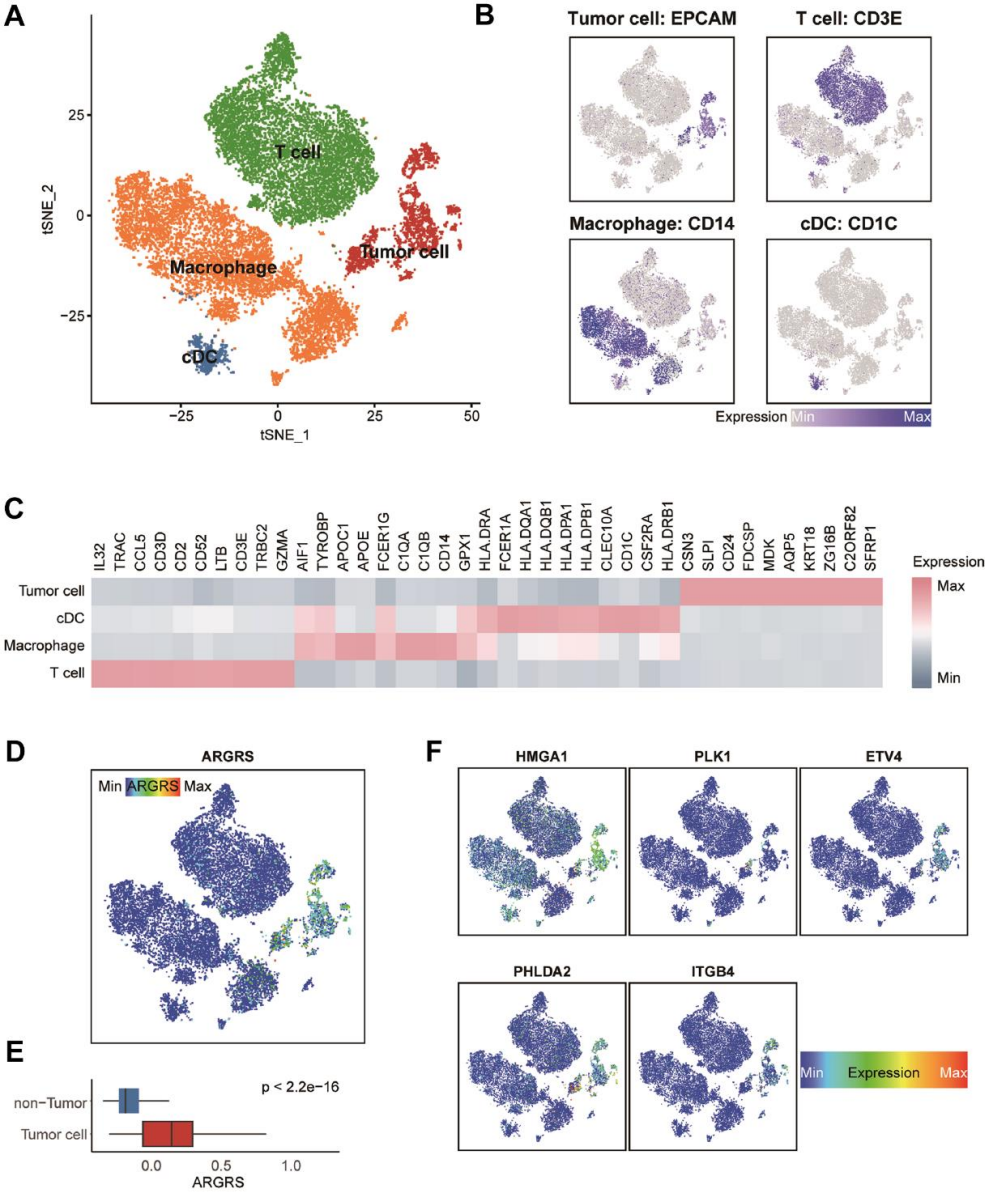
**Transcriptomic clustering of NSCLC patients from GSE148071 dataset.** (**A**) Marker-based cell type identification analysis allowed the prediction of four broad cell types across all profiled single cells. (**B**) Expression levels of cell type signatures overlaid on the t-SNE representation. EPCAM for tumor cells, CD3E for T cells, CD14 for macrophages and CD1C for cDC. (**C**) Gene expression heatmap of top-10 cell type-specific marker genes as measured by Wilcoxon rank-sum test. (**D**) ARGRS overlaid on the t-SNE representation. (**E**) Boxplot showing the levels of ARGRS between tumor and non-tumor cells. Horizontal lines in the boxplots represent the median, the lower and upper hinges correspond to the first and third quartiles, and the whiskers extend from the hinge up to 1.5 times the interquartile range from the hinge. (**F**) Expression levels of the five genes in the prognostic model overlaid on the t-SNE representation.

To further clarify the tumor-specificity of signatures in the model, scRNA-seq datasets were include. A scRNA-seq dataset contained five NSCLC patients were collected and integrated firstly (Supplementary Figure 4A, 4B). After quality control, integration and unsupervised clustering, the cells were divided into 19 clusters (Supplementary Figure 4B). Then, based on the expression distribution of canonical signatures of different cell types, the cells were identified as tumor cells, T cells, macrophages and cDC (Figure 4A–4C). Then, we explored the distribution and expression levels of five crucial genes. Results showed that compared with non-tumor cells, tumor cells had higher levels of ARGRS (Figure 4D, 4E). Besides, the signatures in the model were specific highly expressed in the tumor cells (Figure 4F), suggesting the tumor-specificity of the five genes, and implying that the risk score can represent the status of tumor cells.

Additionally, given of the heterogeneity among NSCLC patients, another scRNA-seq dataset was used to further confirm the results found in the GSE150660 dataset. After preprocessing, a total of 9,563 individual cells of the GSE148071 cohort were passed quality control. Subsequently, unsupervised clustering and cell annotation were performed, and these cells were classified into tumor cells, T/NK, macrophages, and fibroblasts (Supplementary Figure 5A–5D). Consistently, tumor cells had higher levels of ARGRS (Supplementary Figure 5E, 5F) and the genes in the prognostic model (Supplementary Figure 5G), further confirming the tumor cell-specificity of the five genes.

### Patients with higher ARGRS exhibit low immune infiltration

**Figure 5:**
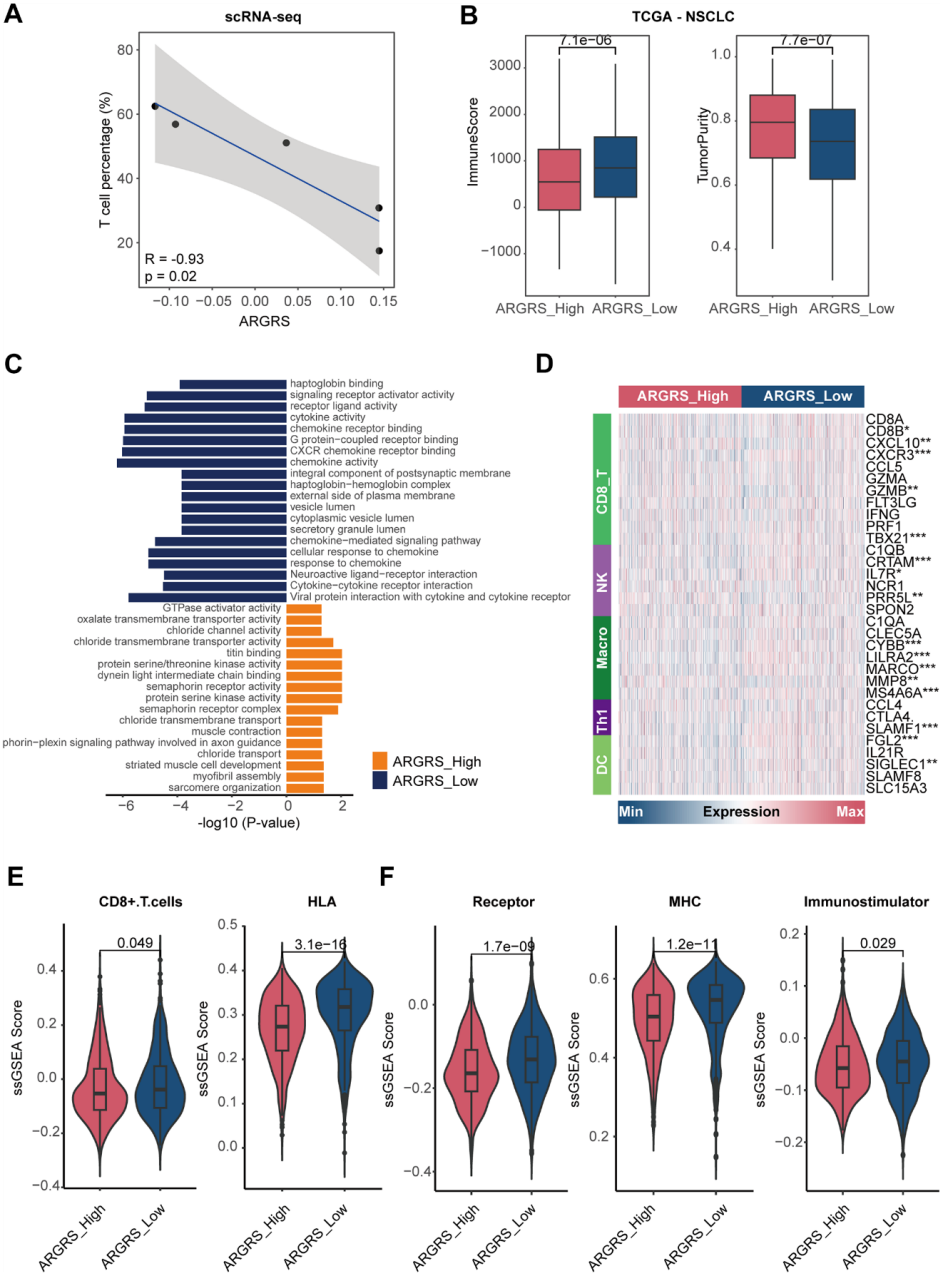
**Patients with higher ARGRS exhibit low immune infiltration.** (**A**) The correlation between the fraction of T cells and ARGRS levels at the single-cell level. X-axis: the fraction of T cells of each NSCLC patient. Y-axis: the median levels of ARGRS of each NSCLC patient. (**B**) Boxplot showing the levels of ImmuneScore (left) and TumorPurity (right) between the ARGRS-high and low groups in the TCGA NSCLC cohort. (**C**) GO and KEGG analyses of genes up-regulated in the ARGRS-high and low groups in the TCGA NSCLC cohort. (**D**) Heatmap showing the levels of immunological markers between ARGRS-high and low groups in the TCGA NSCLC cohort. (**E**, **F**) Comparison of immunological signatures between ARGRS-high and low groups in the TCGA NSCLC cohort.

Having observed the existence of many immune cells in the NCLSC patients at the high dimensional datasets, we next explored the correlation between ARGRS and the fraction of each cell type. Notably, the ARGRS was negatively correlated with the fraction of T cells at the single-cell level (R^2^ = -0.93, p = 0.02, Figure 5A). Also, in the TCGA cohort, patients with higher ARGRS had lower levels of ImmuneScore but higher levels of tumor purity (Figure 5B), consistent with the results found in the single-cell datasets. In addition, a functional analysis of up-regulated genes in ARGRS-low group was highly related to immunological processes, such as cytokine activity, chemokine receptor binding and activity, and cytokine-cytokine receptor interaction (Figure 5C). Meanwhile, compared with the ARGRS-high group, the ARGRS-low group expressed higher transcriptional levels of immunological signatures, such as the biomarkers of CD8+T cells and NK (Figure 5D and Supplementary Figure 6). Also, the ARGRS-low group had higher fraction of CD8+T cells, and enriched some immune activation characteristics, such as the molecules of HLA, MHC, and immunostimulatory (Figure 5E, 5F). To be sum up, combined with the results found at the single-cell and omics transcriptional datasets, we found that ARGRS was negatively correlation with the fraction of T cells, and patients with the ARGRS-high phenotype exhibited low immune infiltration.

In another independent cohort, we also found the similar results. ARGRS was negatively correlated with ImmuneScore (R^2^ = -0.32, p < 0.0001), but positively with tumor purity (R^2^ = 0.36, p < 0.0001, Supplementary Figure 7A–7C). Patients with the ARGRS-low phenotype had higher enrichment scores of immune-related characteristics, such as receptors and MHC molecules (Supplementary Figure 7D). Additionally, patients in the ARGRS- low groups also had higher levels of the immune-related signaling pathways and subpopulations (Supplementary Figure 7E), indicating activate immunological status of patients with the ARGRS- low phenotype. To further investigate the potential factors mediating the formation of inflamed or dessert TME, we deconstructed the interactions among cell populations. Results showed that compared with ARGRS- tumor cells, ARGRS+ tumor cells presented significantly more specific interactions with immune subpopulations (Supplementary Figure 8A). ARGRS+ tumor cells can communicate with T cell via many ligand-receptors (Supplementary Figure 8B), such as TIGIT-NECTIN2 [45] and LGALS9-CD44 [46], which were involved in suppressing tumor T cell infiltration, and promoted the cell state transition of immune cells towards a more immunosuppressive and exhaustive status, indicating the potential mechanism in mediating the dessert TME in the ARGRS-high group.

### Patients with higher ARGRS were resistance to immunotherapy

Previous studies reported that the reactivity of immunotherapy was affected by the immunological status of patients. To be specific, hot tumors, featured by T-cell inflammation, were more sensitive to immunotherapies, while cold tumors are resistant to many treatments [9, 10]. Combined with the distinct immunological status between the ARGRS-high and low groups, the two groups may have different response of immunotherapy.

**Figure 6:**
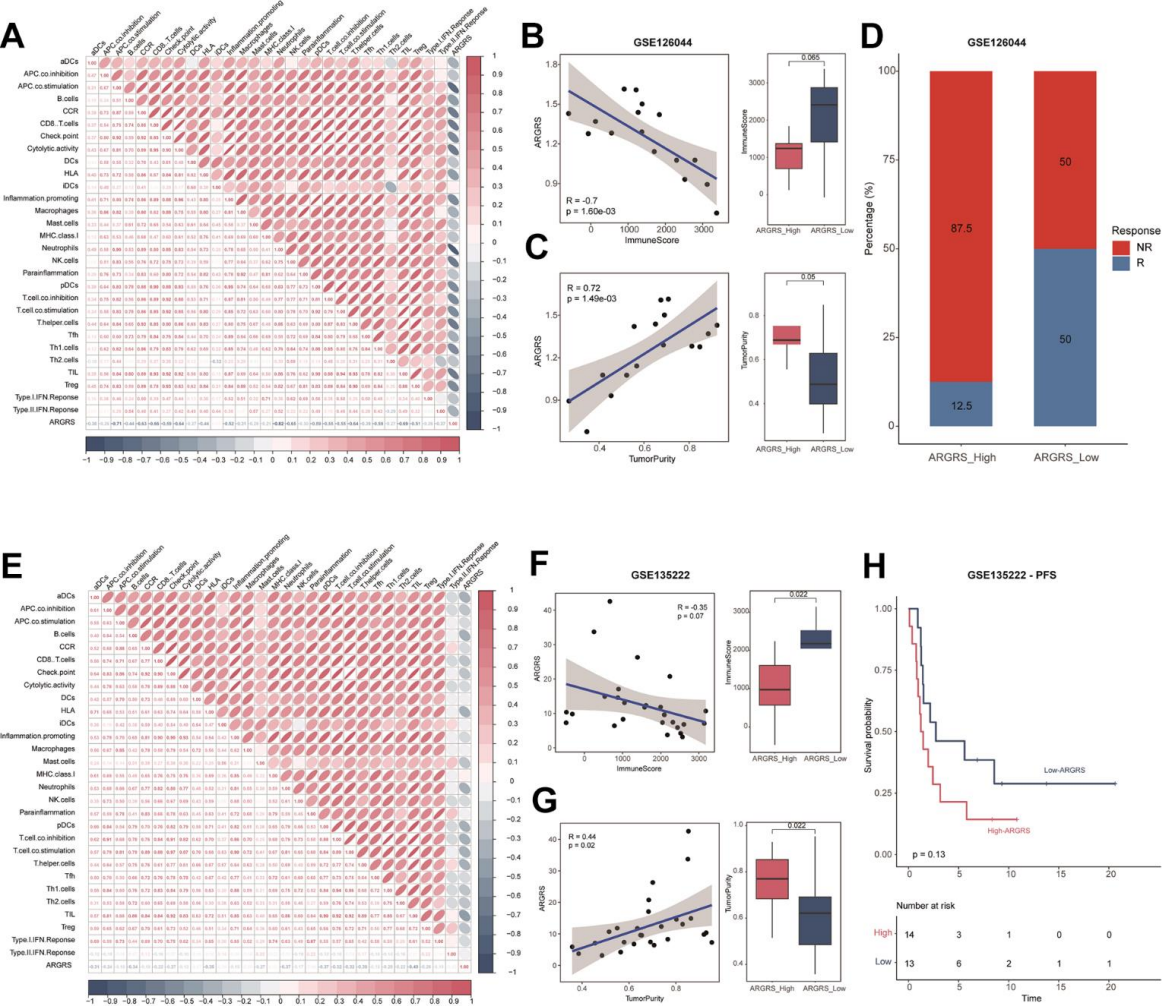
**Patients with higher ARGRS were resistance to immunotherapy.** (**A**) The correlation among 29 immune cell types and immune-related pathways and ARGRS in the GSE126044 cohort. (**B**) Left: Correlation between ARGRS and ImmuneScore in the GSE126044 cohort. Right: Boxplot showing the levels of ImmuneScore between ARGRS-high and low groups. (**C**) Left: Correlation between ARGRS and TumorPurity in the GSE126044 cohort. Right: Boxplot showing the levels of TumorPurity between ARGRS-high and low groups. (**D**) Barplot showing the percentage of immunotherapy responsive and non-responsive NSCLC patients in ARGRS-high and low groups. (**E**) The correlation among 29 immune cell types and immune-related pathways and ARGRS in the GSE135222 cohort. (**F**) Left: Correlation between ARGRS and ImmuneScore in the GSE135222 cohort. Right: Boxplot showing the levels of ImmuneScore between ARGRS-high and low groups. (**G**) Left: Correlation between ARGRS and TumorPurity in the GSE135222 cohort. Right: Boxplot showing the levels of TumorPurity between ARGRS-high and low groups. (**H**) Survival analysis showing the prognostic value of ARGRS in the GSE135222 cohort.

Here, to investigate the immunotherapeutic in the high- and ARGRS-low groups, we downloaded the expression matrix and clinical annotations of NSCLC patients in the cohorts received immunotherapy. In accordance with results above, ARGRS was negatively correlated with almost all immunological characteristics in the GSE126044 dataset (Figure 6A). Also, patients with the ARGRS-high phenotype had higher levels of ImmuneScore, but low tumor purity (Figure 6B, 6C). Besides, patients in the ARGRS-high group were centralized in the NR group (Figure 6D), indicating the immunotherapeutic resistance of them. Consistently, analysis of another immunotherapy cohort also showed the same results. Patients in the ARGRS-high group exhibit lower immune infiltration but higher tumor purity (Figure 6E–6G). Moreover, NSCLC patients in the ARGRS-high group were more likely to recurrence or progress after immunotherapy than the ARGRS-low group (Figure 6H), further supporting the immunotherapeutic resistance of them.

### HMGA1 was the representative biomarker for NSCLC

**Figure 7:**
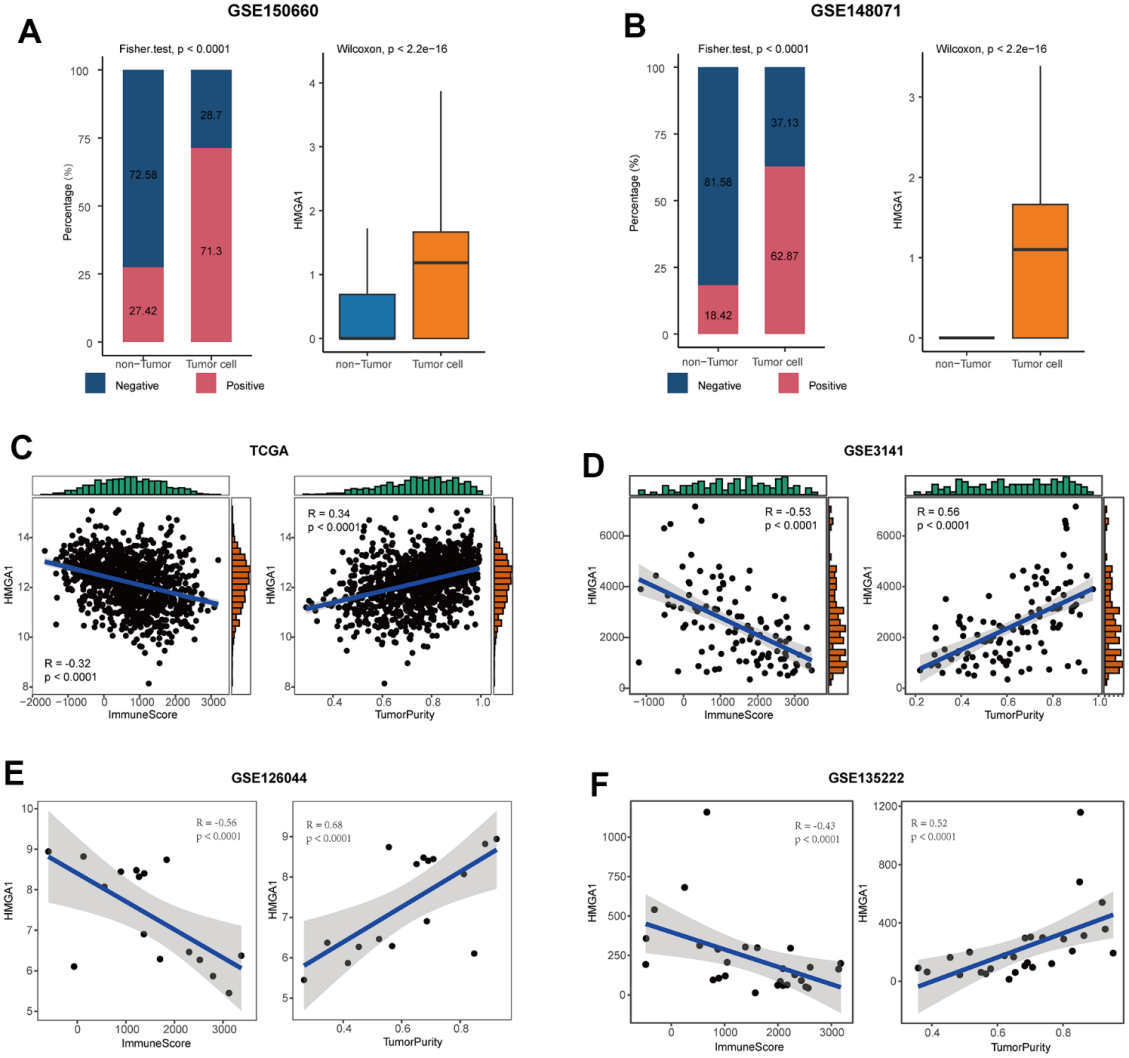
**HMGA1 was the representative biomarker.** (**A**) Right: bar plot showing the expressed fraction of HMGA1 between the non-tumor and tumor cells in the GSE150660 cohort. Left: comparison of the expression levels of HMGA1 between the non-tumor and tumor cells in the GSE150660 cohort. (**B**) Right: bar plot showing the expressed fraction of HMGA1 between the non-tumor and tumor cells in the GSE148071 cohort. Left: comparison of the expression levels of HMGA1 between the non-tumor and tumor cells in the GSE148071cohort. (**C**–**F**) Correlations between HMGA1 expression and ImmuneScore and tumor purity in (**C**) the TCGA, (**D**) the GSE3141, (**E**) the GSE126044, and (**F**) the GSE135222 cohorts.

Next, we chose a representative biomarker from the five genes in the model for further experiments. Firstly, we summarized the expressed fraction of these genes at the single-cell levels. Compared with other genes in the model, tumor cells had obviously transcriptional levels and the highest expressed fraction of HMGA1 (Figure 7A, 7B, and Supplementary Figure 9A, 9B). Besides, at the high-resolution dataset, compared with other four genes, HMGA1 showed the most significant negative correlation with T cell inflamed and positive with tumor cells (Supplementary Figure 10). A Additionally, HMGA1 expressed was negatively correlated with ImmuneScore, but positively with tumor purity in multiple independent cohorts (Figure 7C–7F), suggesting the guidance of HMGA1 in distinguishing the hot and cold NSCLC tumors.

**Figure 8:**
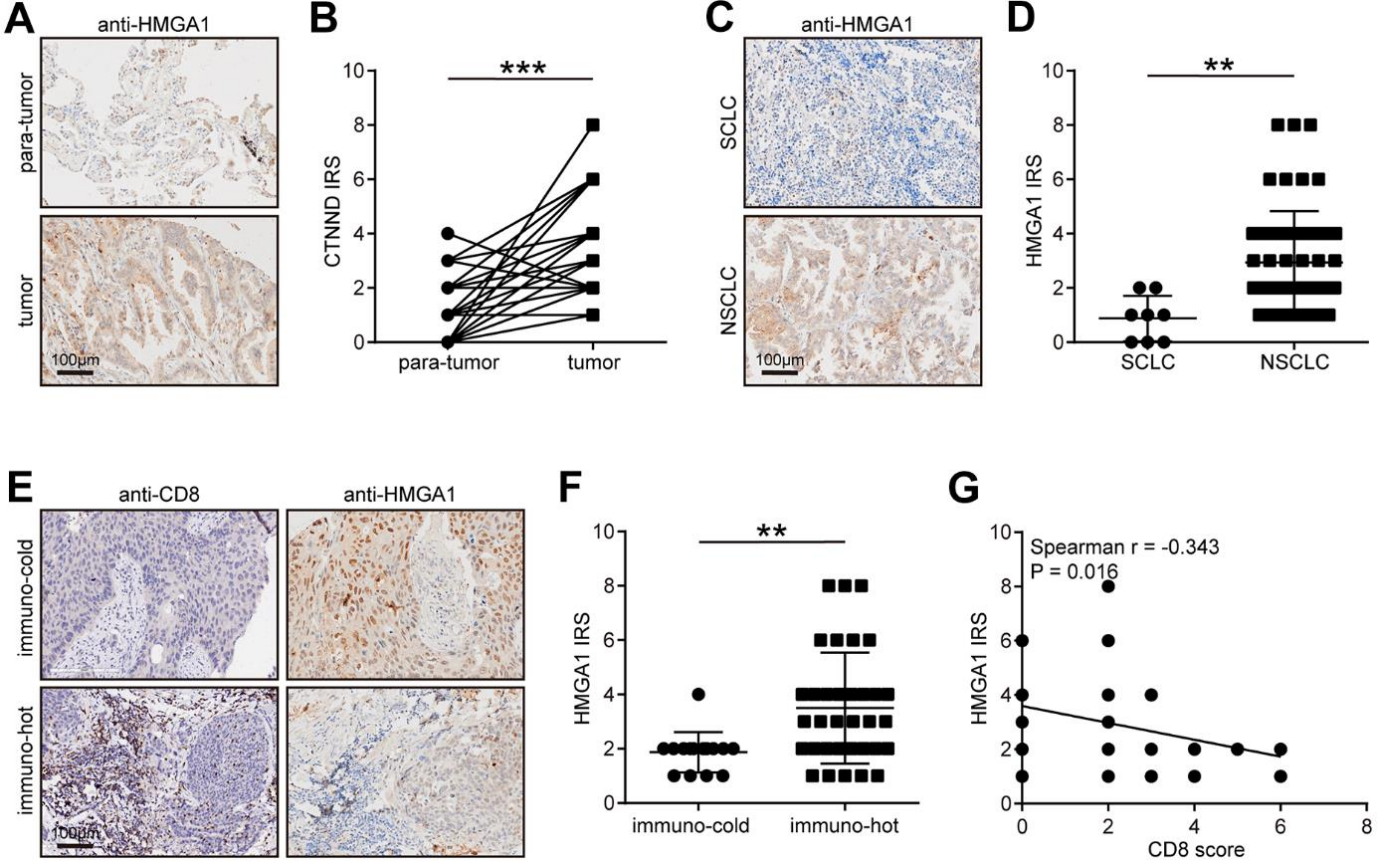
**Validation of CTNND1 expression and its correlations with the TME features in the in-house cohort.** (**A**, **B**) Representative images uncovering the expression of HMGA1 in para-tumor and tumor tissues and quantitative analysis. (**C**, **D**) Representative images uncovering the expression of HMGA1 in NSCLC and SCLC tumor tissues and quantitative analysis. (**E**, **F**) Representative images uncovering the expression of HMGA1 in immuno-cold and immuno-hot tumor tissues and quantitative analysis. (**G**) Correlation between HMGA1 expression and CD8 score.

Next, we verified the expression pattern of HMGA1 in the in-house cohort. We found that HMGA1 expression was significantly higher in tumor tissues than in para-tumor tissues (Figure 8A, 8B), and HMGA1 was specifically expressed in NSCLC (Figure 8C, 8D). We also classified NSCLC into immune-hot and cold tumors based on the CD8 scores. Besides, HMGA1 was significantly reduced in immune-hot tumors (Figure 8E, 8F) and negatively correlated with CD8 scores (Figure 8G). Overall, the negative correlation between HMGA1 expression and T cell infiltration can be verified in the in-house cohort, which greatly improves the confidence of the public cohort results.

## DISCUSSION

Nowadays, with the more profound of molecular and microenvironment characteristics of NSCLC, immunotherapy strategies, such as immune checkpoint inhibitors, has changed first-line treatment of advanced NSCLC, as we all know. Meanwhile, more than 10 indications for immunotherapies in NSCLC clinical practice has been approved by Chinese official organization [47–49].

Even while some patients have benefitted from this new therapy strategy in terms of survival, some individuals are still unable to get lasting relief. Tumor cell apoptosis can result in medication resistance, which in turn affects tumor cell survival in the bloodstream, which is essential for the development of metastasis. Jin et al. found that PLAG1 GDH1 axis promotes apoptotic resistance and tumor metastasis in LKB1-deficient malignancies via CamKK2 AMPK signaling pathway [50]. According to research, CPT1A-mediated fatty acid oxidation can encourage the spread of colorectal cancer cells by impairing the process of cell death [51]. Anoikis has been extensively investigated in relation to tumor growth and metastasis, but little is known about how it alters the tumor immune milieu and hence mediates tumor progression. We concentrate on the part anoikis play in the microenvironment of the tumor and investigate if anoikis can mediate the regulation of immunization, altering the growth and metastasis of the tumor.

We identified tumor-specific anoikis-related genes in NSCLC patients and used these genes to build a predictive model. Patients with higher ARGRS exhibited worse clinical outcomes, showing that ARGRS plays a role in the malignancy of NSCLC. Furthermore, using single-cell transcriptional datasets, we demonstrated that, when compared to non-tumor cells, tumor cells showed greater levels of ARGRS and signature expression in the prognostic model. Notably, ARGRS was negatively linked with the proportion of T cells at the single-cell level, meaning that ARGRS-high NSCLC patients were more likely to have the TME with minimal immune infiltration. Further investigation revealed that the results from the independent NSCLC cohorts were compatible with the findings at the high dimensionality datasets. Patients in the ARGRS-high group, in particular, showed lower levels of immune features but higher tumor purity.

Given of the important role of immunological status of TME in immunotherapeutic response [9, 10, 52], we further investigated the responses of the ARGRS-high and low groups in immunotherapy cohorts. Approximately 87.5% of patients in the ARGRS-high group did not achieve remission after immunotherapy, and were more likely to recurrence or progress, suggesting the immunotherapeutic resistance of these patients. There is now evidence that NSCLC patients can benefit from immunotherapy. However, limited by the inadequate research of characteristics to distinguish the inflamed and desert TME, numerous patients received immunotherapy without obtaining effective results. Therefore, we performed a comprehensive study on explore the ARGs and delved into the prognosis and immune microenvironment characteristics of NSCLC. Additionally, given the regulating upstream feature of ARGRS, targeting it can activate the patient’s immune system coincident with tumor cell killing.

In addition, we further validated the expression and immuno-correlations of HMGA1 in NSCLC in the in-house cohort. We found that HMGA1 was highly expressed in tumor tissues and enriched in immuno-cold tumors, indicating that HMGA1 could shape immuno-cold TME and promote immunosuppression. In fact, reversal of HMGA1-mediated immunosuppression could improve hepatocellular carcinoma therapy [53]. In the term of molecular mechanisms, HMGA1 acted as a crucial regulator of tumor-promoting macrophage recruitment by activating NF-κB-CCL2 signaling and also regulated PD-L1 expression to promote immunosuppression [54, 55].

## CONCLUSIONS

In this study, we investigated NSCLC-specific anoikis-related genes and constructed a prognostic model. Patients with higher ARGRS had worse clinical outcomes. Besides, we proved that the ARGRS and signatures in the models were highly expressed on tumor cells, compared with the non-tumor cells at the single-cell level. Notably, we found that the ARGRS was negatively correlated with the fraction of T cells. And other independent cohorts also showed the same results. To be specific, the ARGRS-high group had low immune infiltration, while the ARGRS-low group showed more active immunological status. Furthermore, in the immunotherapy cohort, patients who did not achieve remission or had tumor progression after immunotherapy were enriched in the ARGRS-high group, suggesting that patients with the ARGRS-high phenotype were more likely to be resistant to immunotherapy. To sum up, we constructed an anoikis-related gene model, and explained the relationship between ARGRS and immunological status, which can help to develop more personalized and precise treatment strategies in clinical practice.

## Supplementary Material

Supplementary Figures
