# Supplementary material for: Anoikis-related gene signature is associated with immune infiltration and predicts the prognosis of non-small cell lung cancer
Source: Aging (Albany NY). 2024 Feb 7;16(3):2908–33. doi: 10.18632/aging.205522 (PMC10911374; doi:10.18632/aging.205522)
Supplement: Supplementary Figures [file aging-16-205522-s001.pdf]

SUPPLEMENTARY FIGURES

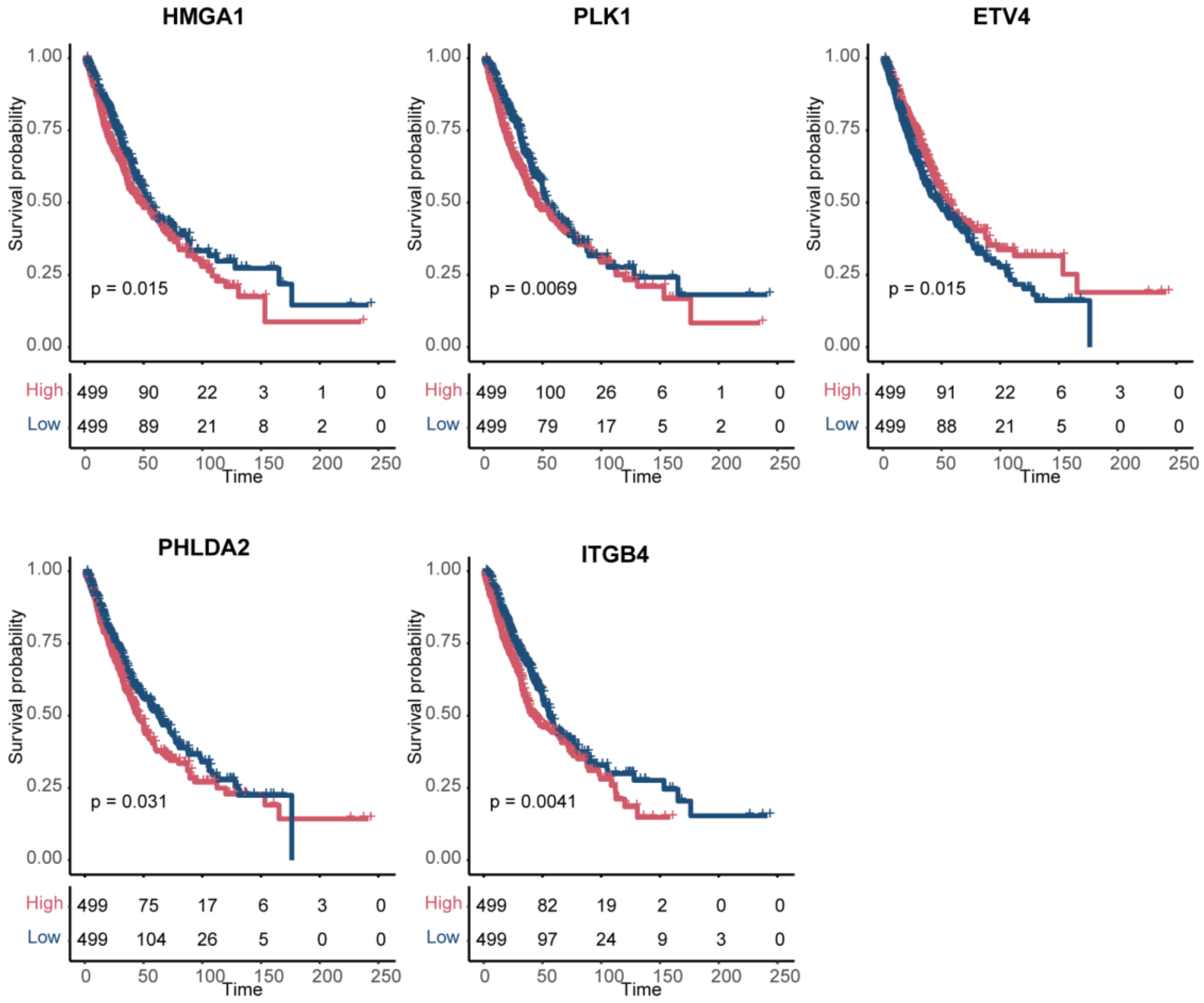

Supplementary Figure 1. Survival analysis showing the prognostic value of the five genes in the prognostic model in the TCGA cohort.

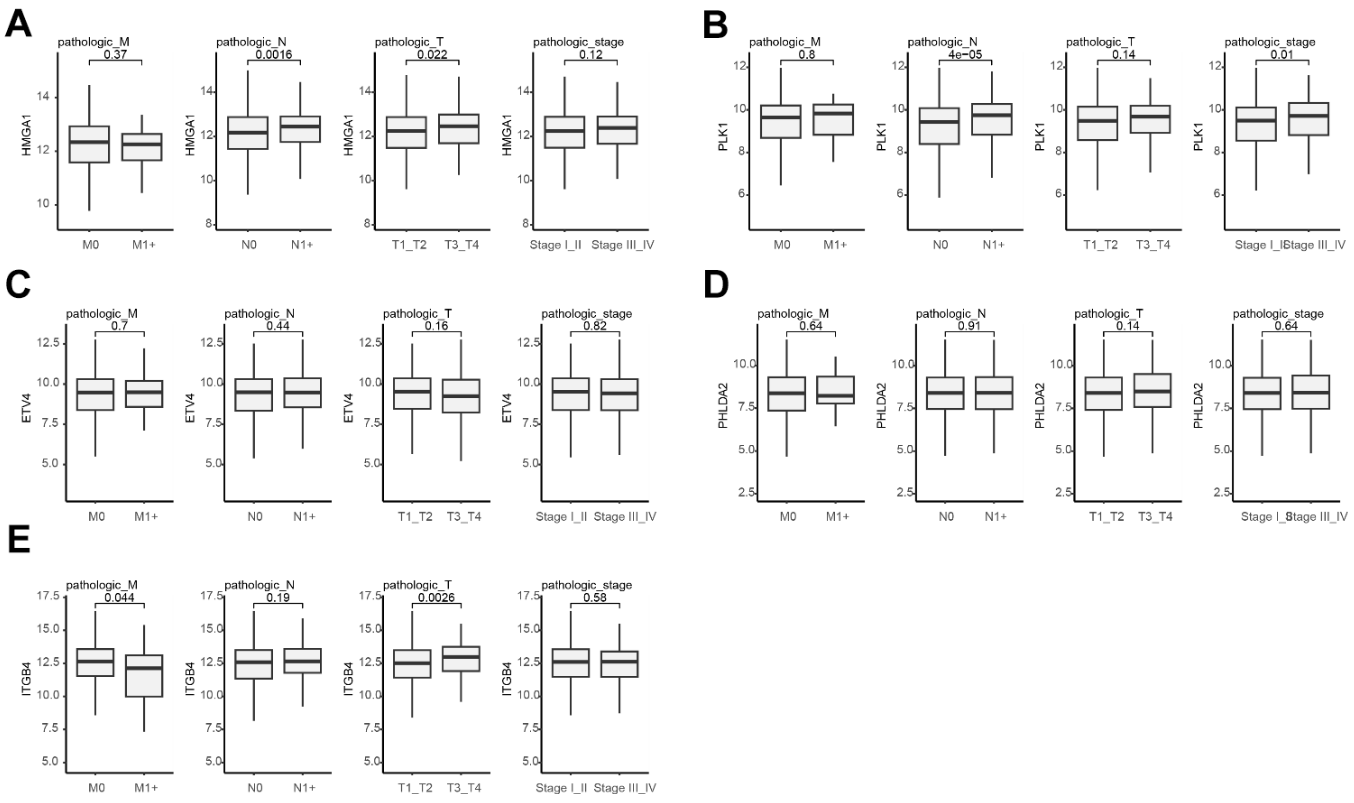

Supplementary Figure 2. Comparing the gene expression of signatures in the prognostic model among patients with different pathological characteristics. (**A**–**E**) Boxplot showing the expression levels of HMGA1 (**A**), PLK1 (**B**), ETV4 (**C**), PHLDA1 (**D**), and ITGB4 (**E**) among patients with different pathological characteristics.

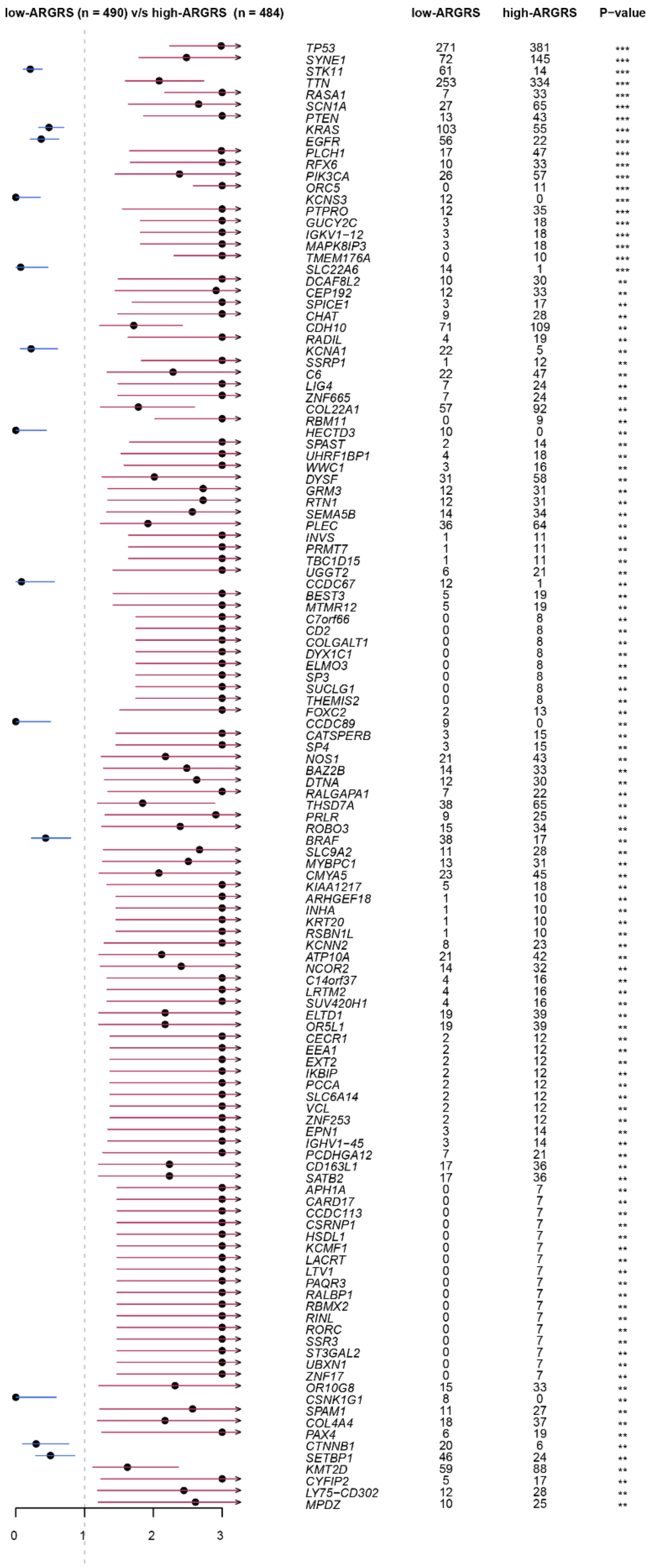

Supplementary Figure 3. Comparison of mutant alterations between the high- and low-ARGRS groups. Genes with p-value < 0.01 were displayed.

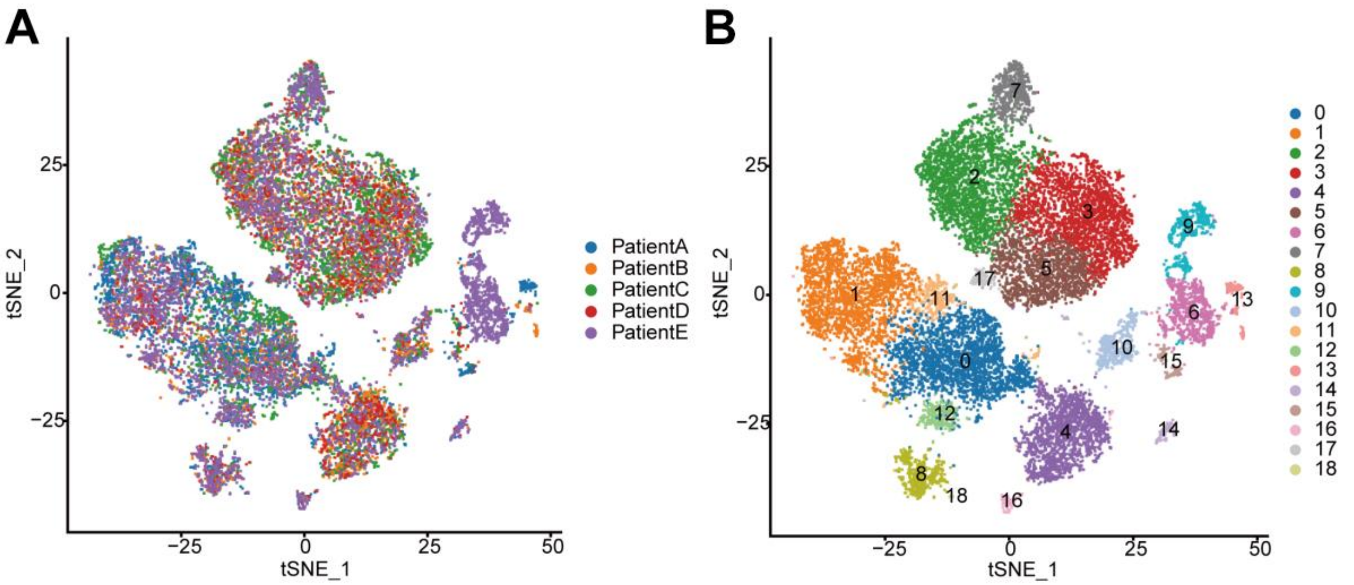

Supplementary Figure 4. Unsupervised clustering of five NSCLC patients from the GSE150660 dataset at the single-cell level. (**A**) The distribution of NSCLC patients across all profiled single cells. (**B**) Unsupervised clustering of all profiled single cells.

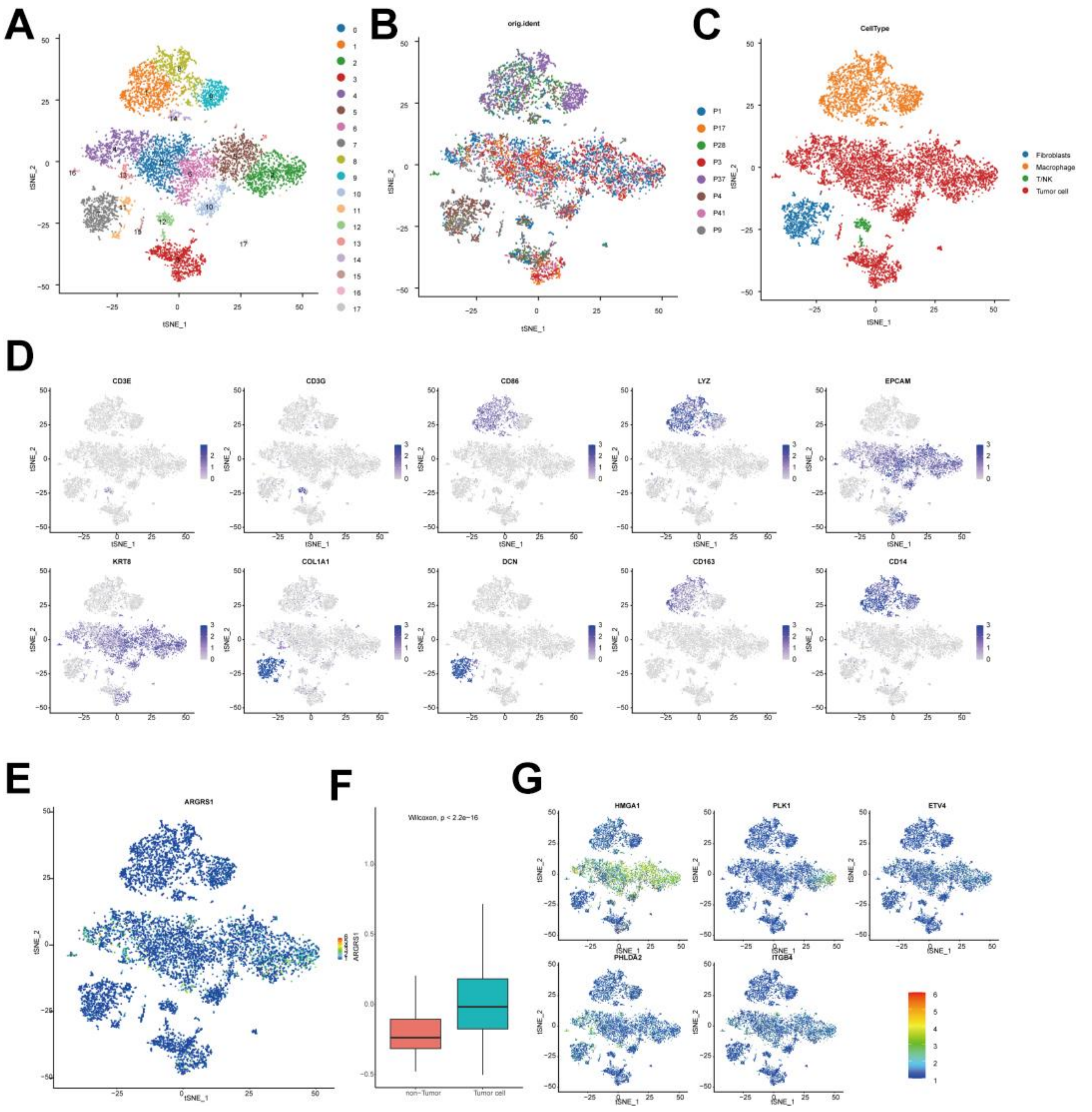

Supplementary Figure 5. Transcriptomic clustering of NSCLC patients in the GSE148071 cohort. (**A**)Unsupervised clustering of 9,563 cells. (**B**) The distribution of NSCLC patients across all profiled single cells. (**C**) Marker-based cell type identification analysis allowed the prediction of four broad cell types across all profiled single cells. (**D**) Expression levels of cell type signatures overlaid on the t-SNE representation. EPCAM and KRT8 for tumor cells, CD3E and CD3G for T cells, CD86, LYZ, CD163 and CD14 for macrophages, COL1A1 and DCN for fibroblasts. (**E**) ARGRS overlaid on the t-SNE representation. (**F**) Boxplot showing the levels of ARGRS between tumor and non-tumor cells. Horizontal lines in the boxplots represent the median, the lower and upper hinges correspond to the first and third quartiles, and the whiskers extend from the hinge up to 1.5 times the interquartile range from the hinge. (**G**) Expression levels of the five genes in the prognostic model overlaid on the t-SNE representation.

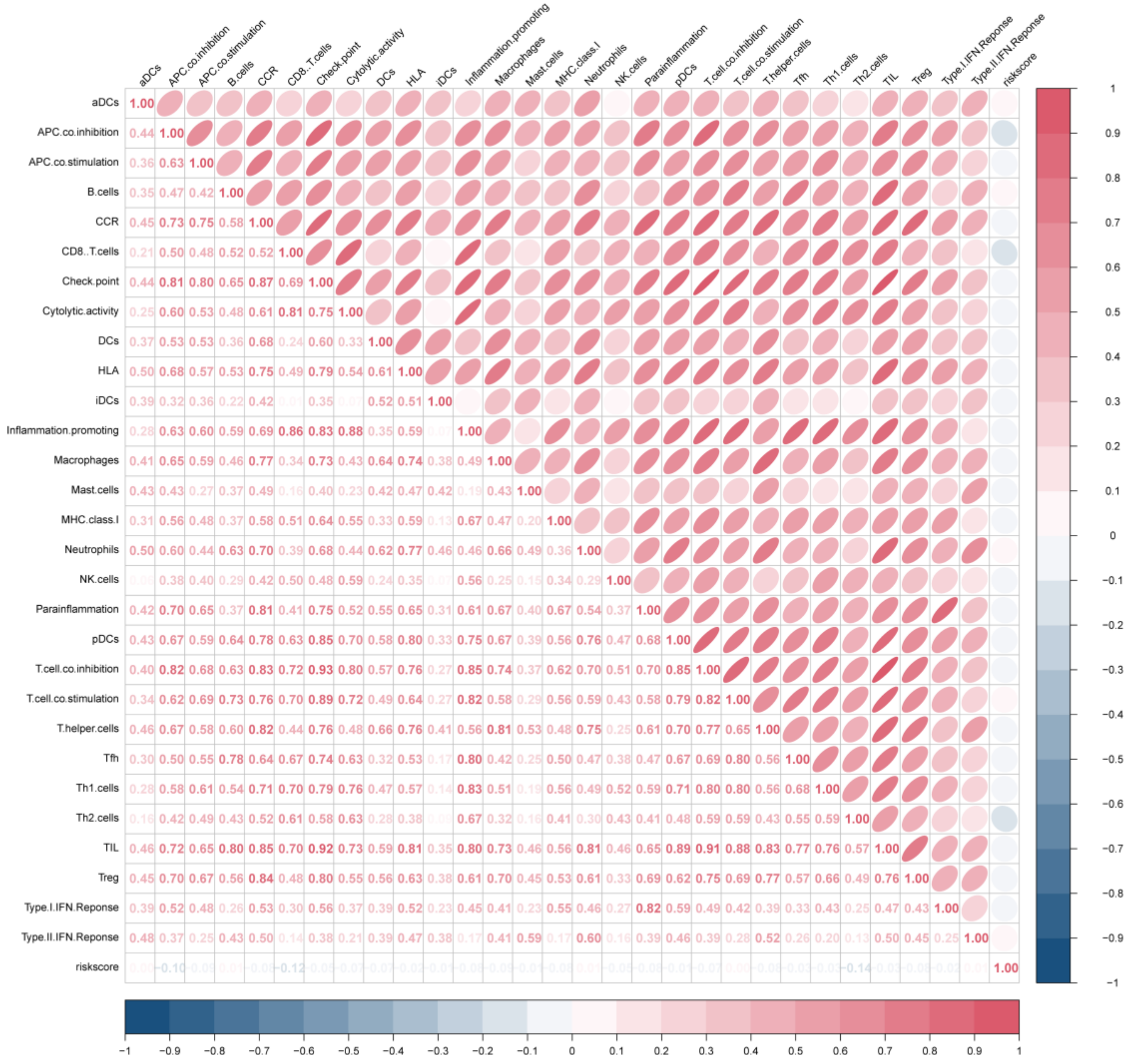

Supplementary Figure 6. The correlation among 29 immune cell types and immune-related pathways and ARGRS in the TCGA cohort.

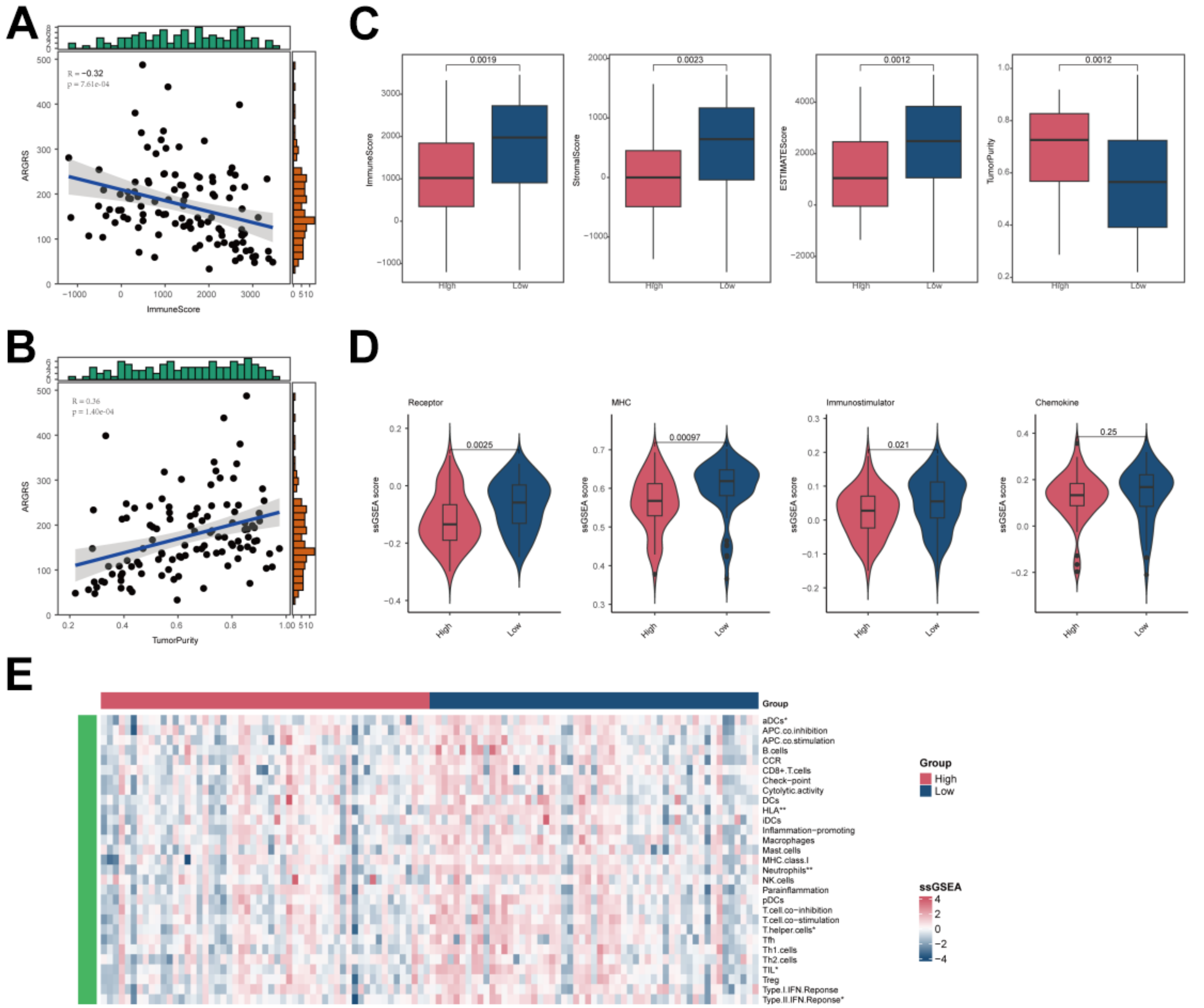

Supplementary Figure 7. Correlation between immunological characteristics and ARGRS in the GSE3141 cohort. (**A**, **B**) Correlation between ARGRS and ImmuneScore (**A**) and tumor purity (**B**). (**C**) Comparison of ImmuneScore, StromalScore, ESTIMATEScore, and tumor purity between ARGRS-high and low groups. (**D**) Comparison of the enrichment of receptors, MHC molecules, Immunostimulator, and Chemokine between ARGRS-high and low groups. (**E**) Heatmap showing the enrichment scores of 29 immunological characteristics.

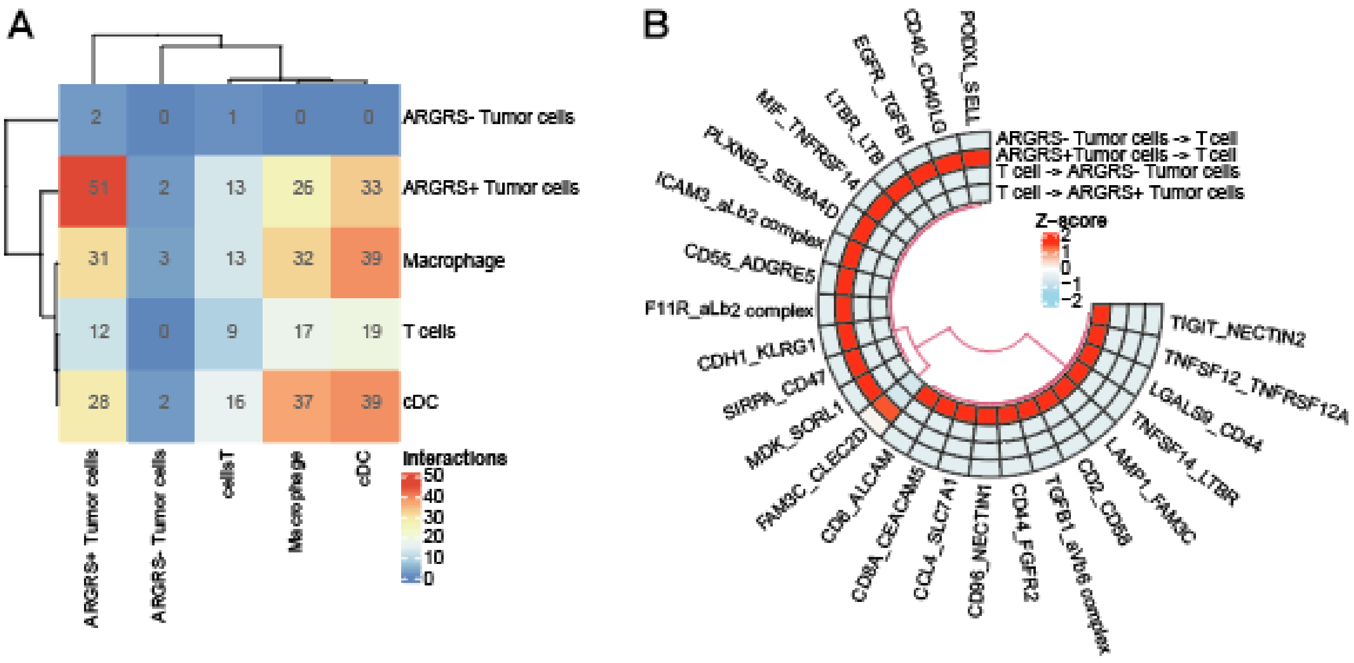

Supplementary Figure 8. Cell-cell communications among cell types in NSCLC (GSE150660). (**A**) Heatmap showing the interaction numbers among cell types. (**B**) Heatmap showing the ligand-receptors between tumor (ARGRS+ and ARGRS-) and T cells.

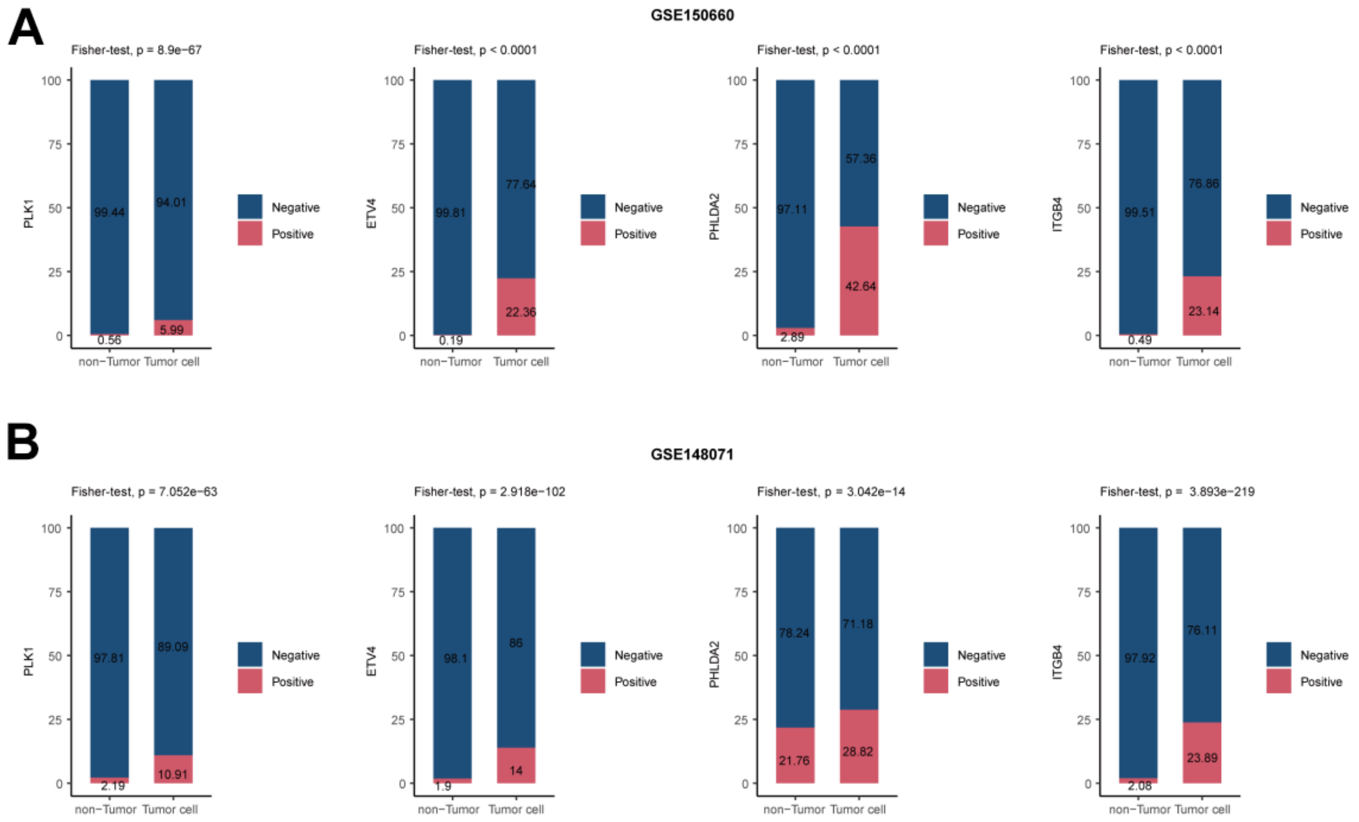

Supplementary Figure 9. Comparison of expressed fraction of genes in the model between non-tumor and tumor cells. (**A**) Bar plot showing the expressed fraction of PLK1, ETV4, PHLDA2, ITGB4 between the non-tumor and tumor cells in the GSE150660 cohort. (**B**) Bar plot showing the expressed fraction of PLK1, ETV4, PHLDA2, ITGB4 between the non-tumor and tumor cells in the GSE148071 cohort.

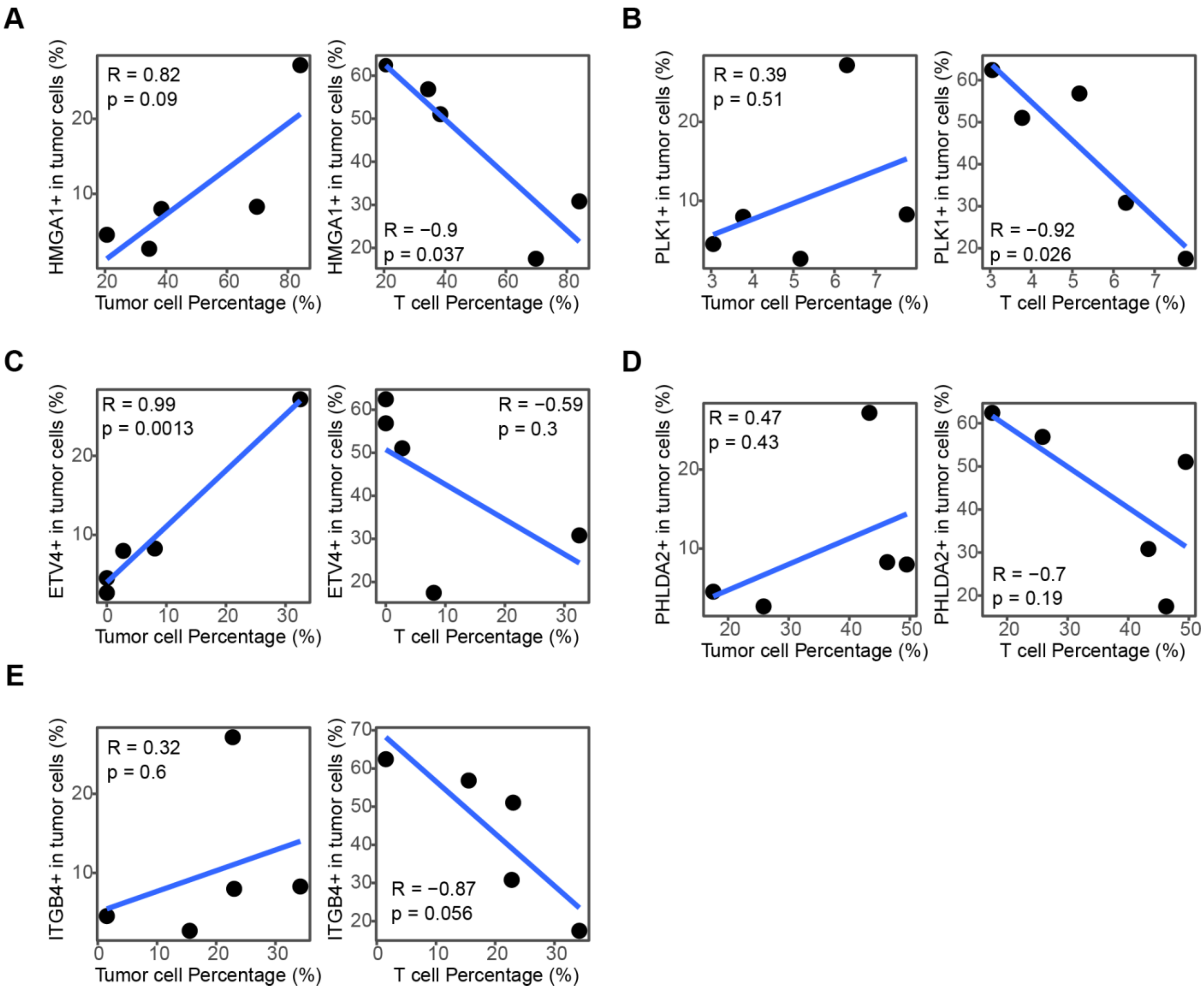

Supplementary Figure 10. Correlation between expressed fractions of genes in the model and tumor/T cell percentage in GSE150660. (**A**) Correlation between HMGA1+ cells in tumor cells and tumor (left) and T cell percentage (right) in GSE150660 dataset. (**B**) Correlation between PLK1+ cells in tumor cells and tumor (left) and T cell percentage (right) in GSE150660 dataset. (**C**) Correlation between ETV4+ cells in tumor cells and tumor (left) and T cell percentage (right) in GSE150660 dataset. (**D**) Correlation between PHLDA2+ cells in tumor cells and tumor (left) and T cell percentage (right) in GSE150660 dataset. (**E**) Correlation between ITGB4+ cells in tumor cells and tumor (left) and T cell percentage (right) in GSE150660 dataset.
